# Incidence of Interstitial Lung Disease in Patients With Rheumatoid Arthritis Treated With Biologic and Targeted Synthetic Disease-Modifying Antirheumatic Drugs

**DOI:** 10.1001/jamanetworkopen.2023.3640

**Published:** 2023-03-20

**Authors:** Matthew C. Baker, Yuhan Liu, Rong Lu, Janice Lin, Jason Melehani, William H. Robinson

**Affiliations:** 1 Division of Immunology and Rheumatology, Department of Medicine, Stanford University, Stanford, California; 2 Quantitative Sciences Unit, Division of Biomedical Informatics Research, Department of Medicine, Stanford University, Stanford, California; 3 Now with Gilead Sciences, Gilead Sciences Inc, Foster City, California; 4 VA Palo Alto Health Care System, Palo Alto, California

## Abstract

**Importance:**

Current data are lacking regarding the risk of biologic and targeted synthetic disease-modifying antirheumatic drug (b/tsDMARD) use on the development of interstitial lung disease (ILD) in patients with rheumatoid arthritis (RA).

**Objective:**

To determine the risk of developing ILD in patients with RA undergoing treatment with different b/tsDMARDs.

**Design, Setting, and Participants:**

Retrospective cohort study using claims data from the Optum Clinformatics Data Mart between December 2003 and December 2019. Adult patients with RA, 1 year or more of continuous enrollment, treatment with a b/tsDMARD of interest, and without preexisting ILD were included. Data were analyzed from October 2021 to April 2022.

**Exposures:**

New administration of adalimumab, abatacept, rituximab, tocilizumab, or tofacitinib.

**Main Outcomes and Measures:**

Crude incidence rates (IRs) for the development of ILD were calculated. The risk of ILD across different b/tsDMARDs was compared using Cox-regression models. A sensitivity analysis using a prevalent new-user cohort design compared patients treated with tofacitinib and adalimumab.

**Results:**

A total of 28 559 patients with RA (mean [SD] age 55.6 [13.7] years; 22 158 female [78%]) were treated with adalimumab (13 326 patients), abatacept (5676 patients), rituximab (5444 patients), tocilizumab (2548 patients), or tofacitinib (1565 patients). Crude IRs per 1000 person-years for ILD were 3.43 (95% CI 2.85-4.09) for adalimumab, 4.46 (95% CI 3.44-5.70) for abatacept, 6.15 (95% CI 4.76-7.84) for rituximab, 5.05 (95% CI 3.47-7.12) for tocilizumab, and 1.47 (95% CI 0.54-3.27) for tofacitinib. After multiple adjustments, compared with patients treated with adalimumab, patients treated with tofacitinib had a lower risk of ILD (adjusted hazard ratio [aHR] 0.31; 95% CI, 0.12-0.78; *P* = .009). In a prevalent new-user cohort analysis, patients treated with tofacitinib had 68% reduced risk of ILD compared with adalimumab (aHR 0.32; 95% CI 0.13-0.82; *P* < .001). In an adjusted model, there was a 69% reduced risk of ILD in patients treated with tofacitinib compared with patients treated with adalimumab.

**Conclusions and Relevance:**

In this retrospective cohort of patients with RA, patients treated with tofacitinib had the lowest incidence of ILD compared with patients treated with all bDMARDs evaluated, and patients treated with tofacitinib had a reduced risk of ILD compared with patients treated with adalimumab after adjusting for important covariates. Additional prospective studies are needed to better understand the role tofacitinib may play in preventing ILD in patients with RA. These results, while significant, should be interpreted with caution given the fairly small sample size of the tofacitinib group.

## Key Points

**Question:**

Is the risk of developing interstitial lung disease (ILD) in patients with rheumatoid arthritis (RA) modified by treatment with different biologic or targeted synthetic disease-modifying antirheumatic drugs (b/tsDMARDs)?

**Findings:**

In this cohort study,a 4 reduced incidence of ILD was observed in 28 559 patients with RA treated with tofacitinib compared with all classes of bDMARDs, and in an adjusted model, there was a 69% reduced risk of ILD in patients treated with tofacitinib compared with patients treated with adalimumab.

**Meaning:**

Given the reduced risk in ILD among patients with RA treated with tofacitinib, these data suggest future prospective studies with tofacitinib to prevent RA-ILD should be considered.

## Teaser

This cohort study evaluates the incidence of interstitial lung disease in patients with rheumatoid arthritis who have been treated with biologic and targeted synthetic disease-modifying antirheumatic drugs.

## Introduction

Clinically significant interstitial lung disease (ILD) occurs in roughly 10% of patients with rheumatoid arthritis (RA).^1,2^ There are limited data on the pathogenesis of RA-ILD; risk factors include smoking, rheumatoid factor or anti-cyclic citrullinated peptide autoantibodies, and genetic variants including *MUC5B*, *HLA-B54*, *HLA-B40*, and HLA-DQ1B*060.^3,4^ Clinicians must determine the best therapy to control a patient’s joint disease, while at the same time minimize the potential risk of pulmonary toxic effects in these patients with already compromised lung function. Current data are lacking regarding the risk of biologic and targeted synthetic disease-modifying antirheumatic drug (b/tsDMARD) use on the development of ILD in patients with RA.

Early reports suggest tumor necrosis factor-α (TNF-α) inhibitor use in patients with RA-ILD worsened underlying ILD.^5^ Subsequent studies did not support these concerns, and similar rates of ILD were demonstrated with other classes of bDMARDs.^6,7,8^ Theoretically, b/tsDMARDs could induce or worsen ILD through idiosyncratic reactions, tissue injury, or susceptibility to infection, or they may provide a therapeutic effect given the likely harmful role that inflammatory cytokines play in ILD pathogenesis.^6^ Currently, the risks associated with TNF-α inhibitors in patients with RA-ILD are uncertain, and whether other b/tsDMARDs are safer also remains unclear. The objective of this nationwide, retrospective study was to estimate the incidence of ILD in patients with RA treated with different b/tsDMARDs and to compare the risk of developing ILD between patients treated with different b/tsDMARDs and adalimumab (the most commonly prescribed TNF-α inhibitor for RA in our data set).

## Methods

### Data Source and Study Population

Data used were from the Optum Clinformatics Data Mart Database, a deidentified database derived from a large, adjudicated claims data warehouse, from December 1, 2003, to December 31, 2019. This study adhered to the Strengthening the Reporting of Observational Studies in Epidemiology (STROBE) reporting guidelines for cohort studies. Due to the deidentified nature of the data set, this study was exempted from review and the requirement for informed consent by the Stanford University institutional review board.

Patients aged 18 years or older with RA based on 2 or more *International Classification of Diseases, Ninth Revision (ICD-9)* (before October 1, 2015) or *International Statistical Classification of Diseases and Related Health Problems, Tenth Revision (ICD-10)* (after October 1, 2015) diagnosis codes (eTable 1 in Supplement 1) separated by 14 or more days receiving new administration of adalimumab (the most commonly prescribed TNF-α inhibitor), abatacept, rituximab, tocilizumab (the most commonly prescribed interleukin-6 inhibitor), or tofacitinib (the most commonly prescribed Janus kinase inhibitor) for 180 days or more (≥2 doses for rituximab) were included.^9^ The study index date was the date of first prescription/administration of the b/tsDMARD. Patients were required to have 1 or more years continuous enrollment before the index date. Patients were excluded if they had a preexisting diagnosis of ILD according to the presence of 1 or more *ICD-9* or *ICD-10* code (eTable 1 in Supplement 1).

### Outcome Ascertainment

The primary end point was incident ILD, which was determined using published methods demonstrating a sensitivity of 73.2%, specificity of 98.2%, and positive predictive value of 78.5%.^10^ Briefly, incident ILD was determined according to either (1) pulmonologist diagnosis of ILD according to 2 *ICD-9* OR *ICD-10* codes for ILD separated by 30 days or more, or (2) 2 *ICD-9 *or *ICD-10* codes for ILD separated by 30 days or more (nonpulmonologist diagnosed) and a *Current Procedural Terminology* code for a computed tomography scan of the chest between 7 and 180 days before the ILD diagnosis and either pulmonary function tests or a lung biopsy between 7 and 180 days before the ILD diagnosis. For both groups, other ILD had to have been excluded on or after the last ILD diagnosis code date (eTable 2 and eTable 3 in Supplement 1). The date of ILD diagnosis was based on the date of the first *ICD* code for ILD. Patients were followed up until discontinuation of the qualifying b/tsDMARD (plus 90 days to account for outcomes occurring shortly after discontinuation, which may have been the reason for treatment cessation [plus 270 days for rituximab]), addition of a new biologic, end of the study period, disenrollment from the data set, or the primary outcome (incident ILD).

### Statistical Analysis

Baseline demographics are presented as mean (SD) or frequencies (percentage) and compared between cohorts by analysis of variance and χ^2^ tests. Crude incidence rates (IRs) of ILD across treatment groups are presented as events per 1000 person-years. Cox proportional hazard models were used to assess the hazard ratio (HR) and 95% CI of developing ILD among patients with RA receiving different treatments after adjusting for age, sex, race and ethnicity (reported in the database as Asian, Black, Hispanic, White, or unknown), education, geographical region, Charlson comorbidity score (calculated using data from 1 year before to 1 year after the index date), outpatient visit frequency (calculated using data during the entire follow-up period), and concomitant immunosuppressive medication use, with adalimumab as the reference group. Race and ethnicity were assessed because they may play a role in the risk of developing RA and in the treatments offered. Kaplan-Meier curves were created to plot survival probabilities over the follow-up time stratified by the exposure group.

Missing data for the categorical variables are reported in Table 1 as “unknown.” For the continuous variables, there were no missing data. Statistical analyses were conducted using R version 4.1.1 (R Project for Statistical Computing), and Charlson comorbidity scores were calculated using the icd package. The exactci and fmsb packages were used for calculating the 95% CI for IRs and incidence rate ratio (IRR). The coxme package was used for mixed effects Cox proportional hazard models. All 95% CI and *P* values were based on 2-sided hypothesis tests, where *P* < .05 was considered statistically significant. Data were analyzed from October 2021 to April 2022.

**Table 1.** Baseline Characteristics of Patients With Rheumatoid Arthritis by Treatment Group

Characteristic	No. (%)					
	Total cohort (N = 28 559)	Adalimumab (n = 13 326)	Abatacept (n = 5676)	Rituximab (n = 5444)	Tocilizumab (n = 2548)	Tofacitinib (n = 1565)
Age, mean (SD), y	55.6 (13.7)	52.7 (13.1)	57.3 (13.4)	59.4 (14.1)	57.1 (14.2)	57.6 (12.5)
Sex						
Female	22158 (77.6)	9735 (73.1)	4785 (84.3)	4228 (77.7)	2116 (83.0)	1294 (82.7)
Male	6401 (22.4)	3591 (26.9)	891 (15.7)	1216 (22.3)	432 (17.0)	271 (17.3)
Race and ethnicity						
Asian	669 (2.3)	312 (2.3)	135 (2.4)	119 (2.2)	55 (2.2)	48 (3.1)
Black	2825 (9.9)	1291 (9.7)	541 (9.5)	545 (10.0)	282 (11.1)	166 (10.6)
Hispanic	3388 (11.9)	1499 (11.2)	720 (12.7)	673 (12.4)	319 (12.5)	177 (11.3)
White	20196 (70.7)	9520 (71.4)	3998 (70.4)	3772 (69.3)	1798 (70.6)	1108 (70.8)
Unknown	1481 (5.2)	704 (5.3)	282 (5.0)	335 (6.2)	94 (3.7)	66 (4.2)
Education						
Less than 12th grade	117 (0.4)	61 (0.5)	21 (0.4)	20 (0.4)	12 (0.5)	11 (0.7)
High school	7068 (24.7)	3310 (24.8)	1394 (24.6)	1336 (24.5)	622 (24.4)	403 (25.8)
Less than bachelor’s degree	15612 (54.7)	7133 (53.5)	3140 (55.3)	3055 (56.1)	1429 (56.0)	850 (54.3)
Bachelor’s degree	4796 (16.8)	2339 (17.6)	942 (16.6)	823 (15.1)	424 (16.6)	268 (17.1)
Unknown	966 (3.4)	483 (3.6)	179 (3.2)	210 (3.9)	61 (2.4)	33 (2.1)
Region						
North Central	6884 (24.1)	3224 (24.2)	1364 (24.0)	1374 (25.2)	567 (22.3)	352 (22.5)
Northeast	2298 (8.0)	1031 (7.7)	404 (7.1)	541 (9.9)	188 (7.4)	134 (8.6)
South	13543 (47.4)	6529 (49.0)	2748 (48.4)	2282 (41.9)	1215 (47.7)	748 (47.8)
West	5797 (20.3)	2528 (19.0)	1147 (20.2)	1235 (22.7)	567 (22.3)	320 (20.4)
Unknown	37 (0.1)	14 (0.1)	13 (0.2)	12 (0.2)	11 (0.4)	11 (0.7)
Charlson comorbidity score						
0	555 (1.9)	495 (3.7)	17 (0.3)	22 (0.4)	13 (0.5)	12 (0.8)
1-2	17709 (62.0)	9376 (70.4)	3427 (60.4)	2354 (43.2)	1549 (60.8)	1000 (63.9)
3-4	5656 (19.8)	2144 (16.1)	1260 (22.2)	1359 (25.0)	584 (22.9)	308 (19.7)
5-6	2422 (8.5)	767 (5.8)	542 (9.5)	754 (13.9)	225 (8.8)	134 (8.6)
>6	2217 (7.8)	544 (4.1)	430 (7.6)	955 (17.5)	177 (6.9)	111 (7.1)
Yearly outpatient visits, mean (SD), No.	11.9 (8.5)	10.2 (7.3)	13.3 (8.5)	14.2 (10.4)	13.5 (8.8)	11.1 (7.5)
Concomitant medications						
Methotrexate	13009 (45.6)	7096 (53.2)	2520 (44.4)	1769 (32.5)	1010 (39.6)	614 (39.2)
Leflunomide	4065 (14.2)	1619 (12.1)	1071 (18.9)	740 (13.6)	389 (15.3)	246 (15.7)
Azathioprine	806 (2.8)	291 (2.2)	178 (3.1)	252 (4.6)	67 (2.6)	18 (1.2)
Sulfasalazine	2196 (7.7)	1159 (8.7)	437 (7.7)	286 (5.3)	191 (7.5)	123 (7.9)
Glucocorticoids	19510 (68.3)	8805 (66.1)	4041 (71.2)	3663 (67.3)	1893 (74.3)	1108 (70.8)
Prednisone dose, mean (SD), mg/d^a^	6.5 (8.5)	5.6 (6.8)	6.2 (7.0)	9.1 (12.4)	6.8 (8.0)	6.0 (7.5)
Follow-up time, median (IQR), y	1.6 (1.0-2.9)	1.8 (1.1-3.2)	1.7 (1.1-2.9)	1.1 (0.8-2.2)	1.6 (1.0-2.7)	1.6 (1.1-2.8)

^a^ For prednisone dose, all glucocorticoids were converted into the prednisone equivalent.

In the primary analysis, tofacitinib was associated with a reduced risk of ILD compared with adalimumab. Given that tofacitinib is typically used as a third-line agent after adalimumab, there may be inherent biases in this comparison. For this reason, we conducted a sensitivity analysis directly comparing these 2 groups using a prevalent new-user cohort design.^11^ This allowed us to compare the older drug (adalimumab) with the newer drug (tofacitinib) using time-based exposure sets to identify matched participants at the same point in the course of disease (eFigure 1 in Supplement 1). The study cohort included all patients from the base cohort treated with tofacitinib. For each patient treated with tofacitinib, a matched reference patient was identified who did not use tofacitinib and who was instead treated with adalimumab according to time-based exposure sets. Time-based exposure sets were defined as time intervals (±30 days) from the first prescription of adalimumab to the first dose of tofacitinib. Exposed and reference patients were matched 1:3 on time-conditional propensity scores using conditional logistic regression to estimate the propensity to receive tofacitinib. The propensity score was calculated using age, sex, geographical region, Charlson comorbidity score, outpatient visit frequency, and concomitant immunosuppressive medication use. For each exposure set, the time-conditional propensity score of the patient with tofacitinib was within the range of the time-conditional propensity scores of the members of the corresponding exposure set, or the exposure set was eliminated. The reference patients with the 3 closest propensity scores were selected from the exposure sets. Crude IRs were calculated for ILD and mixed effect Cox proportional hazard models with pair-level random intercepts were used to assess the HR and 95% CI of developing ILD among patients with RA treated with tofacitinib compared with adalimumab after adjusting for age, sex, race and ethnicity, education, geographical region, Charlson comorbidity score, outpatient visit frequency, and concomitant immunosuppressive medication use.

## Results

### Patient Characteristics

A total of 28 559 patients with RA (mean [SD] age 56 [13.7] years; 22 158 female [78%]) treated with adalimumab (13 326 patients), abatacept (5676 patients), rituximab (5444 patients), tocilizumab (2548 patients), or tofacitinib (1565 patients) were identified (eFigure 2 in Supplement 1; Table 1). Compared with all other treatment groups, the adalimumab cohort was younger, more frequently male, had a lower Charlson comorbidity score, and had a higher use of concomitant methotrexate.

### Incidence and Risk of ILD

A total of 276 incident cases of ILD occurred during follow-up (119 in the adalimumab cohort, 60 in the abatacept cohort, 62 in the rituximab cohort, 30 in the tocilizumab cohort, and 5 in the tofacitinib cohort) (Table 2). The crude IRs per 1000 person-years for ILD were 3.43 (95% CI, 2.85-4.09) for adalimumab, 4.46 (95% CI, 3.44-5.70) for abatacept, 6.15 (95% CI, 4.76-7.84) for rituximab, 5.05 (95% CI, 3.47-7.12) for tocilizumab, and 1.47 (95% CI, 0.54-3.27) for tofacitinib (Table 2). Patients who received tofacitinib were 69% less likely to develop ILD compared with those treated with adalimumab (aHR 0.31; 95% CI 0.12-0.78; *P* = .009) (Table 2 and Figure). Kaplan-Meier plots showed a notable difference in the survival probabilities for ILD development over the follow-up time between the tofacitinib group and all of the bDMARD groups (eFigure 3 in Supplement 1).

**Table 2.** Incidence and Adjusted HRs of ILD in Patients With Rheumatoid Arthritis by Treatment

Characteristic	Total cohort (N = 28 559)	Adalimumab (n = 13 326)	Abatacept (n = 5676)	Rituximab (n = 5444)	Tocilizumab (n = 2548)	Tofacitinib (n = 1565)
Incident ILD, No. (%)	276 (0.97)	119 (0.89)	60 (1.06)	62 (1.14)	30 (1.18)	5 (0.32)
Person-years, No.	67 087	34 682	13 447	10 074	5492	3392
IR (95% CI)	4.11 (3.65-4.62)	3.43 (2.85-4.09)	4.46 (3.44-5.70)	6.15 (4.76-7.84)	5.05 (3.47-7.12)	1.47 (0.54-3.27)
IRR (95% CI)	NA	1 [Reference]	1.30 (0.95-1.77)	1.79 (1.32-2.44)	1.47 (0.99-2.20)	0.43 (0.18-1.05)
HR (95% CI)						
Crude	NA	1 [Reference]	1.28 (0.94-1.74)	1.71 (1.26-2.33)	1.53 (1.03-2.29)	0.41 (0.17-1.01)
Adjusted^a^	NA	1 [Reference]	0.79 (0.57-1.09)	0.85 (0.61-1.20)	0.99 (0.65-1.50)	0.31 (0.12-0.78)

Abbreviations: HR, hazard ratio; ILD, interstitial lung disease; IR, incidence rate per 1000 person-years; IRR, incidence rate ratio with adalimumab as the reference group; NA, not applicable.

^a^ Adjusted for age, sex, race, education, geographical region, Charlson comorbidity score, outpatient visit frequency, and concomitant immunosuppressive medication use.

**Figure.**
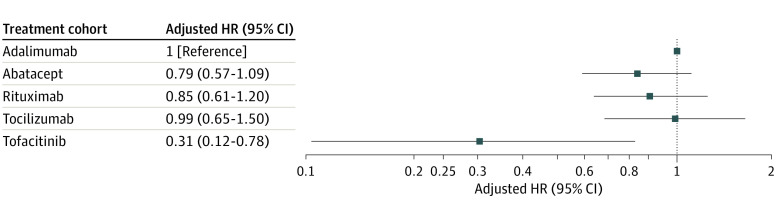
Adjusted Hazard Ratios (HRs) of Interstitial Lung Disease in Patients With Rheumatoid Arthritis by Treatment

### Sensitivity Analysis

Using the prevalent new-user cohort design, after propensity score matching, cohorts of 4677 patients treated with adalimumab and 1559 patients treated with tofacitinib were constructed (mean [SD] age 58 [12.3] years, 2116 were female [83%], 11 outpatient visits per year, roughly 38% methotrexate use for both groups) (Table 3). The incidence rate per 1000 person-years of ILD for patients with RA treated with tofacitinib was 1.48 (95% CI, 0.54-3.28), compared with 4.30 (95% CI, 3.15-5.74) for patients treated with adalimumab (Table 4). In the adjusted model, there was a 68% reduction in risk for the development of ILD in patients treated with tofacitinib compared with patients treated with adalimumab (aHR 0.32; 95% CI, 0.13-0.82; *P* < .001) (Table 4).

**Table 3.** Baseline Characteristics of the Prevalent New-User Cohort of Patients With Rheumatoid Arthritis

Characteristic	No. (%)			SMD
	Total cohort (n = 6236)	Adalimumab (n = 4677)	Tofacitinib (n = 1559)	
Age, mean (SD), y	57.5 (12.3)	57.5 (12.2)	57.5 (12.5)	0.003
Sex				
Female	5166 (82.8)	3878 (82.9)	1288 (82.6)	0.008
Male	1070 (17.2)	799 (17.1)	271 (17.4)	
Race and ethnicity				
Asian	142 (2.3)	94 (2.0)	48 (3.1)	0.089
Black	611 (9.8)	445 (9.5)	166 (10.6)	
Hispanic	698 (11.2)	522 (11.2)	176 (11.3)	
White	4481 (71.9)	3377 (72.2)	1104 (70.8)	
Unknown	304 (4.9)	239 (5.1)	65 (4.2)	
Education				
Less than 12th grade	1078 (17.3)	812 (17.4)	266 (17.1)	0.075
High school	1589 (25.5)	1185 (25.3)	404 (25.9)	
Less than bachelor’s degree	29 (0.5)	24 (0.5)	11 (0.7)	
Bachelor’s degree	3358 (53.8)	2507 (53.6)	845 (54.2)	
Unknown	182 (2.9)	149 (3.2)	33 (2.1)	
Region				
North Central	1401 (22.5)	1048 (22.4)	353 (22.6)	0.024
Northeast	514 (8.2)	380 (8.1)	134 (8.6)	
South	3010 (48.3)	2257 (48.3)	753 (48.3)	
West	1311 (21.0)	992 (21.2)	319 (20.5)	
Charlson comorbidity score				
0	32 (0.5)	24 (0.5)	12 (0.8)	0.026
1-2	4049 (64.9)	3050 (65.2)	995 (63.8)	
3-4	1214 (19.5)	906 (19.4)	308 (19.8)	
5-6	510 (8.2)	376 (8.0)	134 (8.6)	
>6	431 (6.9)	321 (6.9)	110 (7.1)	
Yearly outpatient visits, mean (SD), No.	11.0 (7.8)	10.9 (7.9)	11.1 (7.5)	0.028
Concomitant medications				
Methotrexate	2383 (38.2)	1773 (37.9)	610 (39.1)	0.025
Leflunomide	943 (15.1)	699 (14.9)	244 (15.7)	0.020
Azathioprine	63 (1.0)	45 (1.0)	18 (1.2)	0.019
Sulfasalazine	486 (7.8)	363 (7.8)	123 (7.9)	0.005
Glucocorticoids	4361 (69.9)	3258 (69.7)	1103 (70.8)	0.024
Prednisone dose, mean (SD), mg/d^a^	5.9 (7.0)	5.9 (6.8)	6.0 (7.6)	0.015

Abbreviation: SMD, standardized mean difference.

^a^ For prednisone dose, all glucocorticoids were converted into the prednisone equivalent.

**Table 4.** Incidence of ILD and HRs in the Prevalent New-User Cohort

Characteristic	Adalimumab (n = 4677)	Tofacitinib (n = 1559)
Incident ILD, No. (%)	43 (0.92)	5 (0.32)
Person-years, No.	10 000	3381
IR (95% CI)	4.30 (3.15-5.74)	1.48 (0.54-3.28)
IRR (95% CI)	1 [Reference]	0.34 (0.14-0.87)
HR (95% CI)		
Crude	1 [Reference]	0.33 (0.13-0.84)
Adjusted^a^	1 [Reference]	0.32 (0.13-0.82)

Abbreviations: HR, hazard ratio; ILD, interstitial lung disease; IR, incidence rate per 1000 person-years; IRR, incidence rate ratio.

^a^ Adjusted for age, sex, race and ethnicity, education, geographical region, Charlson comorbidity score, outpatient visit frequency, and concomitant immunosuppressive medication use.

## Discussion

In our large, nationwide retrospective cohort study, the incidence of ILD was lowest among patients with RA treated with tofacitinib. After adjusting for important covariables, our analysis demonstrated a 69% reduction in the risk of developing ILD in patients with RA treated with tofacitinib compared with adalimumab. There was a 21% reduction in the incidence of ILD in patients treated with abatacept compared with adalimumab, which did not reach significance. The adalimumab group had a higher frequency of concurrent methotrexate use compared with all other groups, however recent studies do not demonstrate an increased risk of RA-ILD associated with methotrexate use.^12,13^ In a sensitivity analysis that compared tofacitinib users and adalimumab users at similar time points in their disease course, we similarly found a 68% reduction in the risk of ILD among patients with RA treated with tofacitinib compared with adalimumab.

Few studies have investigated the risk of incident ILD among patients with RA treated with b/tsDMARDs. Our study supports prior literature demonstrating similar incidence rates of ILD among patients with RA treated with various bDMARDs, with our study demonstrating a slightly lower incidence rate for ILD in patients treated with adalimumab and a slightly higher incidence rate in patients treated with abatacept.^14^ In addition, our study included patients treated with tofacitinib, and found tofacitinib to be the only treatment associated with a reduced risk of ILD relative to the other treatments examined. This is in line with data suggesting tofacitinib may be effective for treating ILD associated with dermatomyositis and with reports showing tofacitinib provides benefit in RA-ILD, given its antifibrotic and anti-inflammatory properties.^15,16,17,18,19^ It is also consistent with a post hoc analysis of patients treated with tofacitinib from phase 1, phase 2, phase 3, and phase 4, and long-term extension studies, which demonstrated a comparable incidence rate for ILD of 0.18 per 100 patient-years.^20^

### Strengths and Limitations

Our study has several strengths. We used a large claims database covering patients in a wide geographic area throughout the US. We used a validated claims-based approach to identify ILD, which provides a high specificity and positive predictive value. We performed a sensitivity analysis using a prevalent new-user cohort study design, which allowed us to compare patients treated with adalimumab and tofacitinib at the same time point in their disease course. This helped eliminate bias inherent in this comparison, as patients who switch from adalimumab to tofacitinib may have disease of greater duration.

Our study has several limitations. First, as this is a retrospective study using claims data, there may be residual or unmeasured confounders. We adjusted for baseline characteristics such as age, sex, race and ethnicity, education, geographic region, Charlson comorbidity score, outpatient visit frequency, and concomitant immunosuppressive medication use. Second, we did not have data on certain variables, including smoking, autoantibodies, or genetic risk alleles, which can be associated with RA and ILD. Smoking is associated with ILD, and it is possible that smoking, or another factor that predisposes to the development of ILD, was also a clinical indicator for the selection of a specific b/tsDMARD, and thus we cannot rule out the possibility of residual confounding from the lack of data on several important covariables. Third, the sample size for the tofacitinib group was relatively small, and this may have contributed to a small number of events. However, the follow-up time was similar across all groups, and we used Cox proportional hazard models to investigate the association between time-to-event and use of treatment while controlling for the other baseline characteristics. Fourth, although we used a validated algorithm for identifying incident ILD, this algorithm includes ILD diagnosed by transbronchial or percutaneous biopsy methods, which are less reliable than video-assisted thoracoscopic surgery (VATS) or open lung biopsy. This could have resulted in misclassification of the outcome. Fifth, we excluded patients with preexisting ILD according to *ICD-9* or *ICD-10* codes; however, we did not exclude patients with restrictive lung disease (without ILD). Thus, patients who were already in the process of developing ILD before starting their b/tsDMARD may have been included. Sixth, this study spanned 16 years, and clinical practice likely changed over this time. If coding practices changed as well, this may have influenced the results. Additionally, we cannot determine the degree of medication compliance for any treatment group.

## Conclusions

In this retrospective cohort study of patients with RA, we observed a significant reduction in the risk of developing ILD in patients treated with tofacitinib. These results suggest that treatment with tofacitinib, and perhaps other Janus kinase inhibitors, may provide benefit in reducing the risk of developing RA-ILD. Future prospective studies of tofacitinib in patients with RA to prevent the development of ILD should be considered.

## Notes

Supplement 1. **eTable 1. **Codes Used for Patient Inclusion and Exclusion **eTable 2. **Codes Used in ILD Algorithms **eTable 3. **Codes Used to Exclude Other Causes of ILD **eFigure 1.** Prevalent New-User Study Design With Time-Based Exposure Sets Defined by a 30-Day Interval Surrounding the Timing of Tofacitinib Initiation **eFigure 2.** Cohort Selection **eFigure 3.** Kaplan–Meier Plot for the Risk of ILD During Treatment With Different b/tsDMARDs in Patients With RA

Supplement 2.
Data Sharing Statement
