# Supplementary material for: Incidence of Interstitial Lung Disease in Patients With Rheumatoid Arthritis Treated With Biologic and Targeted Synthetic Disease-Modifying Antirheumatic Drugs
Source: JAMA Netw Open. 2023 Mar 20;6(3):e233640. doi: 10.1001/jamanetworkopen.2023.3640 (PMC10028485; doi:10.1001/jamanetworkopen.2023.3640)
Supplement: Supplement 1. — eTable 1. Codes Used for Patient Inclusion and Exclusion eTable 2. Codes Used in ILD Algorithms eTable 3. Codes Used to Exclude Other Causes of ILD eFigure 1. Prevalent New-User Study Design With Time-Based Exposure Sets Defined by a 30-Day Interval Surrounding the Timing of Tofacitinib Initiation eFigure 2. Cohort Selection eFigure 3. Kaplan–Meier Plot for the Risk of ILD During Treatment With Different b/tsDMARDs in Patients With RA [file jamanetwopen-e233640-s001.pdf]

Supplemental Online Content

Baker MC, Liu Y, Lu R, Lin J, Melehani J, Robinson WH. Incidence of interstitial lung disease in patients with rheumatoid arthritis treated with biologic and targeted synthetic disease-modifying antirheumatic drugs. *JAMA Netw Open*. 2023;6(3):e233640. doi:10.1001/jamanetworkopen.2023.3640

eTable 1. Codes Used for Patient Inclusion and Exclusion
eTable 2. Codes Used in ILD Algorithms
eTable 3. Codes Used to Exclude Other Causes of ILD
eFigure 1. Prevalent New-User Study Design With Time-Based Exposure Sets Defined by a 30-Day Interval Surrounding the Timing of Tofacitinib Initiation
eFigure 2. Cohort Selection
eFigure 3. Kaplan–Meier Plot for the Risk of ILD During Treatment With Different b/tsDMARDs in Patients With RA

This supplemental material has been provided by the authors to give readers additional information about their work.

© 2023 Baker MC et al. *JAMA Network Open.*

**eTable 1.** Codes Used for Patient Inclusion and Exclusion	
**ICD Codes for Rheumatoid Arthritis**	
**ICD-9**	**Diagnosis**
714.0	Rheumatoid arthritis
714.1	Felty's syndrome
714.2	Other rheumatoid arthritis with visceral or systemic involvement
**ICD-10**	**Diagnosis**
M06.00, M06.011, M06.012, M06.019, M06.021, M06.022, M06.029, M06.031, M06.032, M06.039, M06.041, M06.042, M06.049, M06.051, M06.052, M06.059, M06.061, M06.062, M06.069, M06.071, M06.072, M06.079, M06.08, M06.09, M06.0A, M06.811, M06.812, M06.819, M06.821, M06.822, M06.829, M06.831, M06.832, M06.839, M06.841, M06.842, M06.849, M06.851, M06.852, M06.859, M06.861, M06.862, M06.869, M06.871, M06.872, M06.879, M06.88, M06.8, M06.8A, M06.9, M05.60, M05.631, M05.632, M05.639, M05.641, M05.642, M05.649, M05.651, M05.652, M05.659, M05.661, M05.662, M05.669, M05.671, M05.672, M05.679, M05.69, M05.70, M05.711, M05.712, M05.719, M05.721, M05.722, M05.729, M05.731, M05.732, M05.739, M05.741, M05.742, M05.749, M05.751, M05.752, M05.759, M05.761, M05.762, M05.769, M05.771, M05.772, M05.779, M05.79, M05.7A, M05.80, M05.811, M05.812, M05.819, M05.821, M05.822, M05.829, M05.831, M05.832, M05.839, M05.841, M05.842, M05.849, M05.851, M05.852, M05.859, M05.861, M05.862, M05.869, M05.871, M05.872, M05.879, M05.89, M05.8A, M05.9	Rheumatoid arthritis
**ICD Codes for Interstitial Lung Disease**	
**ICD-9**	**Diagnosis**
515.xx	Post-inflammatory pulmonary fibrosis
516.30	Idiopathic interstitial pneumonia not otherwise specified
516.31	Idiopathic pulmonary fibrosis
516.32	Idiopathic non-specific interstitial pneumonitis
516.33	Acute interstitial pneumonitis
516.34	Respiratory bronchiolitis interstitial lung disease
516.35	Idiopathic lymphoid interstitial pneumonia
516.36	Cryptogenic organizing pneumonia
516.37	Desquamative interstitial pneumonia
516.8x	Other specified alveolar and parietoalveolar pneumonopathies
516.9x	Unspecified alveolar and parietoalveolar pneumonopathy

© 2023 Baker MC et al. *JAMA Network Open.*

ICD Codes for Interstitial Lung Disease	
**ICD-10**	**Diagnosis**
J84.1x	Other interstitial pulmonary diseases with fibrosis
J84.10	Pulmonary fibrosis, unspecified
J84.11	Idiopathic interstitial pneumonia
J84.111	Idiopathic interstitial pneumonia, not otherwise specified
J84.112	Idiopathic pulmonary fibrosis
J84.113	Idiopathic non-specific interstitial pneumonitis
J84.114	Acute interstitial pneumonitis
J84.115	Respiratory bronchiolitis interstitial lung disease
J84.116	Cryptogenic organizing pneumonia
J84.117	Desquamative interstitial pneumonia
J84.17	Other interstitial pulmonary diseases with fibrosis in diseases classified elsewhere
J84.170	Interstitial lung disease with progressive fibrotic phenotype in diseases classified elsewhere
J84.178	Other interstitial pulmonary diseases with fibrosis in diseases classified elsewhere
J84.89	Other specified interstitial pulmonary diseases
J84.9	Interstitial pulmonary disease, unspecified

CPT = current procedural terminology; ICD = international classification of diseases.

© 2023 Baker MC et al. *JAMA Network Open.*

eTable 2. Codes Used in ILD Algorithms

Procedure	CPT	ICD-9 CM Procedure	ICD-10 PCS
Lung Biopsy			
Surgical	32095, 32096, 32097, 32602, 32607, 32608	33.20, 33.28, 34.21	0BB30*X-0BB90*X, 0BBB0*X-0BBM0*X
Transbronchial	31628, 31629, 31632	33.27	0BB38*X-0BB98*X, 0BBB8*X-0BBM8*X
Percutaneous	32405	33.26	0BB33*X-0BB93*X, 0BBB3*X-0BBM3*X
Chest computed tomography (CT)			
Chest CT	71250, 71260, 71270	87.41	BB24***
Low dose chest CT	G0297		
CT-angiogram	71275		
Pulmonary function tests (PFTs)			
Spirometry	94010, 94060, 94070, 94150, 94200, 94375	89.37, 89.38	4A09***
Lung volume	94250, 94726, 94727		
Diffusion capacity	94729		

ILD = interstitial lung disease; CPT = current procedural terminology; ICD = international classification of diseases; CM = clinical modification; PCS = procedure coding system. *denotes any code in this position.

Table adopted from: England BR, Roul P, Mahajan TD, Singh N, Yu F, Sayles H, et al. Performance of Administrative Algorithms to Identify Interstitial Lung Disease in Rheumatoid Arthritis. Arthritis Care Res (Hoboken). 2020 Oct; 72(10):1392-1403.

© 2023 Baker MC et al. *JAMA Network Open.*

**eTable 3.** Codes Used to Exclude Other Causes of ILD	
**ICD Codes for Other Causes of ILD to Exclude**	
**ICD-9**	**Diagnosis**
135.x	Sarcoidosis
517.2, 710.1	Systemic sclerosis
710.3-710.4	Myositis
710.0	Systemic lupus erythematosus
495.x	Hypersensitivity pneumonitis
500.x-505.x	Pneumoconioses (including asbestos)
508.1	Radiation pneumonitis
**ICD-10**	**Diagnosis**
D86.x	Sarcoidosis
M34.x	Systemic sclerosis
M33.x	Myositis
M32.x	Systemic lupus erythematosus
J67.x	Hypersensitivity pneumonitis
J60.x-J64.x	Pneumoconioses (including asbestos)
J70.0-J70.1	Radiation pneumonitis

ICD = international classification of diseases; ILD = interstitial lung disease.

Table adopted from: England BR, Roul P, Mahajan TD, Singh N, Yu F, Sayles H, et al. Performance of Administrative Algorithms to Identify Interstitial Lung Disease in Rheumatoid Arthritis. Arthritis Care Res (Hoboken). 2020 Oct; 72(10):1392-1403.

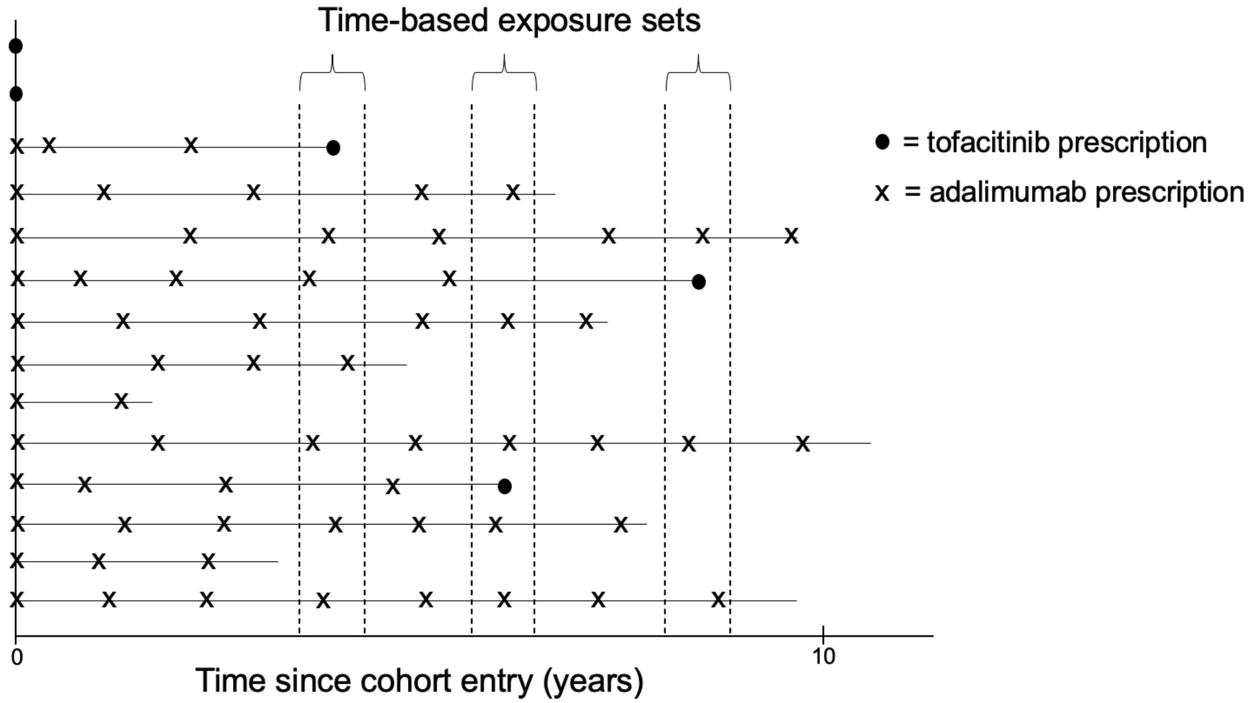

eFigure 1. Prevalent New-User Study Design With Time-Based Exposure Sets Defined by a 30-Day Interval Surrounding the Timing of Tofacitinib Initiation

Figure adopted from: Suissa S, Moodie EE, Dell'Aniello S. Prevalent new-user cohort designs for comparative drug effect studies by time-conditional propensity scores. Pharmacoepidemiol Drug Saf. 2017 Apr; 26(4):459-468.

© 2023 Baker MC et al. *JAMA Network Open.*

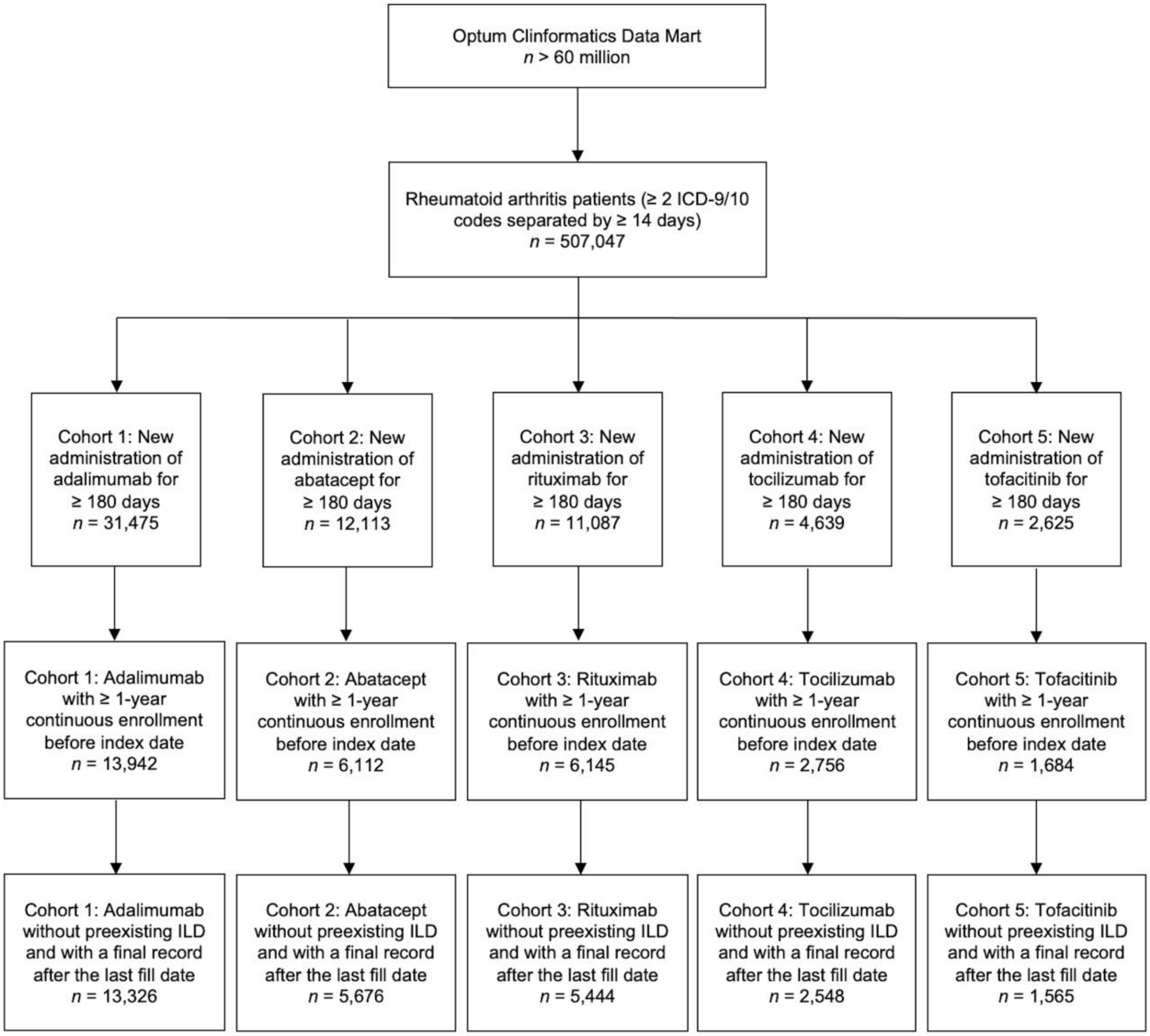

eFigure 2. Cohort Selection

© 2023 Baker MC et al. *JAMA Network Open.*

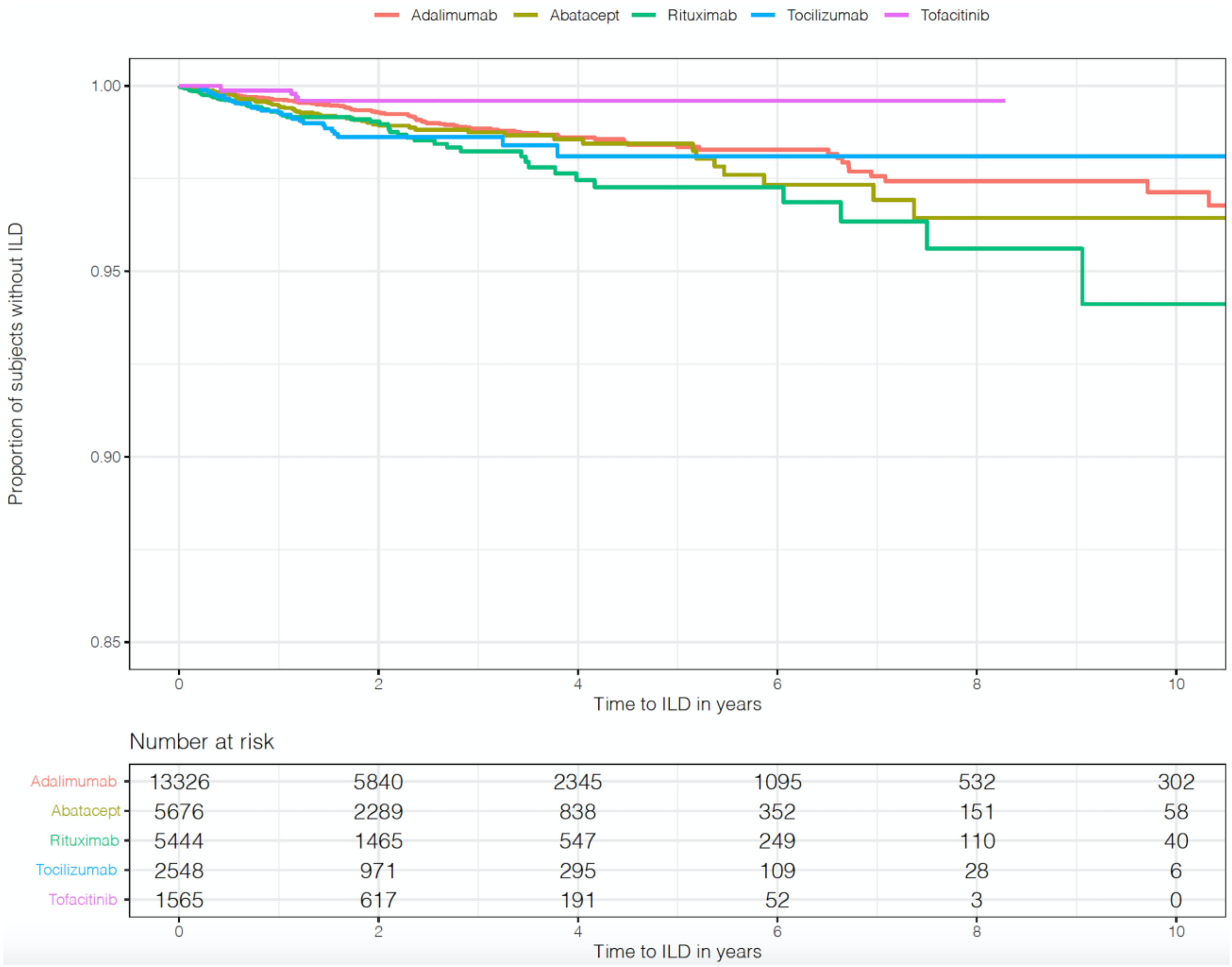

eFigure 3. Kaplan–Meier Plot for the Risk of ILD During Treatment With Different b/tsDMARDs in Patients With RA

© 2023 Baker MC et al. *JAMA Network Open.*
