# Supplementary material for: Incidence of Interstitial Lung Disease in Patients With Rheumatoid Arthritis Treated With Biologic and Targeted Synthetic Disease-Modifying Antirheumatic Drugs
Source: JAMA Netw Open. 2023 Mar 20;6(3):e233640. doi: 10.1001/jamanetworkopen.2023.3640 (PMC10028485; doi:10.1001/jamanetworkopen.2023.3640)
Supplement: Supplement 2. — Data Sharing Statement [file jamanetwopen-e233640-s002.pdf]

Data Sharing Statement

Baker. Incidence of Interstitial Lung Disease in Patients With Rheumatoid Arthritis Treated With Biologic and Targeted Synthetic Disease-Modifying Antirheumatic Drugs. *JAMA Netw Open*. Published March 20, 2023. doi:10.1001/jamanetworkopen.2023.3640

Data
Data available: No
